# The Relationship between Body Mass Index and Incident Diabetes Mellitus in Chinese Aged Population: A Cohort Study

**DOI:** 10.1155/2021/5581349

**Published:** 2021-08-24

**Authors:** M. L. Tang, Y. Q. Zhou, A. Q. Song, J. L. Wang, Y. P. Wan, R. Y. Xu

**Affiliations:** Department of Clinical Nutrition, Ren Ji Hospital, School of Medicine, Shanghai Jiao Tong University, Shanghai 200127, China

## Abstract

**Objectives:**

Previous studies reported that overweight older adults had a lower mortality after cardiovascular diseases attack, indicating being thinner might not always be better. However, there is an ongoing debate about what is the optimal range of body mass index (BMI) for the aged population. We aimed to evaluate the value of BMI for the prediction of incident diabetes mellitus (DM) in the Chinese elderly population.

**Methods:**

A total number of 6,911 Chinese elderly people (4,110 men and 2,801 women, aged 71 ± 6.0 years) were included in this cohort study. BMI was measured at baseline (Jan 1, 2014, to Dec 31, 2014). All the participants were further classified into six groups: <18.5 kg/m^2^, 18.5 to <22.5 kg/m^2^, 22.5 to <25.0 kg/m^2^, 25.0 to <27.5 kg/m^2^, 27.5 to <30.0 kg/m^2^, and ≥30.0 kg/m^2^. Fasting blood glucose (FBG) and glycated hemoglobin A1c (HbA1c) were annually measured during follow-up (Jan 1, 2015-May 31, 2019). DM was confirmed if either FBG ≥ 7.0 mmol/L or HbA1c ≥ 6.5%. We used the Cox proportional hazard regression model to evaluate the association between BMI and the prediction of incident DM.

**Results:**

Comparing individuals with a BMI range of 18.5 to <22.5 kg/m^2^ (reference), the hazard ratio for incident DM was 2.13 (95% CI: 1.54~2.95), 2.14 (95% CI: 1.53~3.00), 3.17 (95% CI: 2.19~4.59), 3.15 (95% CI: 1.94~5.09), and 3.14 (95% CI: 1.94~5.09) for the group with a BMI range of 22.5 to <25.0 kg/m^2^, 25.0 to <27.5 kg/m^2^, 27.5 to <30.0 kg/m^2^, and ≥30.0 kg/m^2^ after adjusting for baseline age, sex, blood pressure, lipid profiles, and eGFR (*P* trend < 0.001), after adjusting for the abovementioned confounders. The association tended to be closer in men and young participants, compared with their counterparts.

**Conclusions:**

High BMI was associated with a high risk of developing DM in the Chinese aged population. Thus, it is optimal for the aged population to maintain their body weight within a reasonable range to prevent chronic diseases.

## 1. Introduction

China has become a leading country with a dramatic number of aged people. According to data from the National Bureau of Statistics of the People's Republic of China, there were 170 million people aged over 65 years by the end of 2019. Population aging deeply affects the age structure of a population and poses challenges to achieving sustainability.

Diabetes mellitus (DM) is a noncommunicable disease affecting approximately one-quarter of people over 65 years old [1], which significantly increases mortality and disability [2], thus, in turn, increases both direct and indirect medical costs [3]. DM and obesity share many common risk factors [4]. Previous research has established that obesity is associated with insulin resistance and decreased insulin sensitivity in the aged population with newly diagnosed type 2 DM [5] and a predictor of incident type 2 DM [6]. A healthy body mass index (BMI) is believed to be helpful to reduce the prevalence of DM in adults. A WHO panel reported that there is an increasing evidence of a high prevalence of type 2 DM as well as cardiovascular disease among Asian population at a lower BMI than the standard cutoff of 25.0 kg/m^2^ [7]. A cohort study, including different ethnic participants, reported that Chinese in general have a lower BMI cutoff than black and white individuals (25.0 vs. 26.0 vs. 30.0 kg/m^2^, respectively), thus, supporting the belief that lower BMI is needed when screening for DM in nonwhite populations [8]. However, there has been little discussion about generating the optimal BMI range for aged population. One retrospective study with 88,305 Japanese aged participants has reported that the optimal BMI cut-off for the prediction of DM was 23.6 kg/m^2^ [9]. Further, existing evidence of the association between BMI and all-cause mortality follows a “U” [10, 11] or “J” curve [12, 13]. This indicates that the phenotype of overweight (BMI ≈ 25.0 ~ 29.9 kg/m^2^) might be more beneficial in aging population [11, 14, 15]. Having said this, it is not clear that the association between BMI and DM in the elderly follows the same pattern. Therefore, we aimed to evaluate the value of BMI for the prediction of incident diabetes mellitus (DM) in 6,911 Chinese aged population with 5 years of follow-up. We proposed that a relatively lower BMI is needed to prevent the risk of DM than a higher BMI.

## 2. Materials and Methods

### 2.1. Study Population

All the participants (≥65 y) were recruited from the local communities who have taken health check-up at Health Management Center, Ren Ji Hospital from January 1, 2014, to May 31, 2019. A total number of 9,902 Chinese aged participants were eligible for the study. BMI was measured on the day of their site visit from January 1 to December 31, 2014. Fasting blood glucose (FBG) and glycated hemoglobin A1c (HbA1c) were measured annually during follow-up (Jan 1, 2015, to May 31, 2019). We excluded participants whose FBG ≥ 7.0 mmol/L, or HbA1c ≥ 6.5%, or with self-reporting DM at baseline (*n* = 2,012). Finally, we excluded participants lost to follow-up after the baseline recruitment (Jan 1 to Dec 31, 2014) (*n* = 979). As a result, a total number of 6,911 Chinese aged population (4,110 men and 2,801 women, aged 71 ± 6.0 years) were included in the study (Supplementary Figure 1). The study protocol was approved by the Ethical Committee of Ren Ji Hospital, School of Medicine, Shanghai Jiao Tong University. As a reidentified study, the signed consent was waived by the ethical committee.

### 2.2. Exposures (BMI)

Body weight and height were measured in light clothes with no shoes at baseline, and BMI was calculated from dividing weight (kilogram) by height squared (meter). All the participants were further classified into six groups: <18.5 kg/m^2^, 18.5 to <22.5 kg/m^2^, 22.5 to <25.0 kg/m^2^, 25.0 to <27.5 kg/m^2^, 27.5 to <30.0 kg/m^2^, and ≥30.0 kg/m^2^ [16].

### 2.3. Outcomes (Incident DM)

Venous blood samples were drawn and transfused into vacuum tubes containing EDTA in the morning after participants fasted overnight for eight hours. FBG was measured by enzyme-linked immunosorbent assay (Roche 701 Bioanalyzer, Roche, UK). HbA1c was measured by a high-performance liquid chromatography method (Variant II automatic glycosylated hemoglobin analyzer, Bio-Rad, America). DM was confirmed if either FBG ≥ 7.0 mmol/L or HbA1c ≥ 6.5% [17].

### 2.4. Assessment of Other Confounders

Blood pressure was measured twice using an automatic blood-pressure meter (HBP-9020, OMRON (China) Co., Ltd.) after participants were seated for at least 10 mins. The average of two measurements was recorded for further analysis. Total cholesterol, triglycerides, high-density lipoprotein cholesterol, and low-density lipoprotein cholesterol were measured as well. The estimating glomerular filtration (eGFR) was calculated using the Chronic Kidney Disease Epidemiology Collaboration 2-level race equation [18]. All the biochemical measurements were performed in the Clinical Laboratory of Ren Ji Hospital.

### 2.5. Statistical Analysis

All statistical analysis was conducted by SAS version 9.4 (SAS Institute, Inc., Cary, NC). Formal hypothesis testing will be Wilcoxon test for rank sum with a significant level of 0.05.

In the current study, we used the Cox proportional hazards regression model to evaluate the association between BMI and the prediction of incident DM in whole group. The person-time of follow-up for each participant was determined from the baseline (January 1, 2014) to either the onset date of DM, loss to follow-up, or the end of follow-up (May 31, 2019), whichever came first.

**Figure 1:**
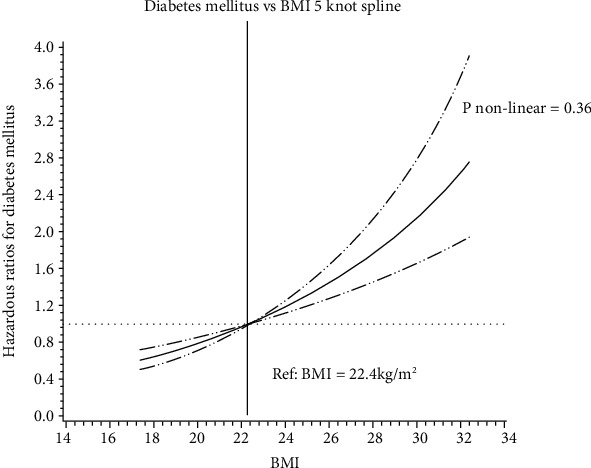
Hazard ratio for diabetes mellitus based on continuous change in BMI based on measurements in 2014 among 6,911 Chinese elderly people. Model was adjusted for age (y), sex, systolic blood pressure (mmHg), diastolic blood pressure (mmHg), total cholesterol (mmol/L), triglyceride (mmol/L), low-density lipoprotein cholesterol (mmol/L), high-density lipoprotein cholesterol (mmol/L), and eGFR (mL/min per 1.73 m^2^). Data were fitted by a restricted cubic spline Cox proportional hazards model. The 95% confidence intervals are indicated by the dashed line.

With the analysis of dose-response trend, more specifically, the continuous variable of the change in BMI was used to fit into a restricted cubic spline model [19] and to obtain a smooth representation of the hazard ratio as a function of the change in BMI adjusted by potential confounders. We used 5 knots defined at the 5^th^, 27.5^th^, 50^th^, 72.5^th^, and 95^th^ percentiles to divide continuous change in BMI into 5 intervals (Figure 1).

We adjusted for potential confounders in different models: model 1, adjusting for age (y) and sex; model 2, adjusting for variables in model 1, and systolic blood pressure (mmHg), diastolic blood pressure (mmHg), total cholesterol (mmol/L), triglyceride (mmol/L), low-density lipoprotein cholesterol (mmol/L), high-density lipoprotein cholesterol (mmol/L), and eGFR (mL/min per 1.73 m^2^); model 3, adjusting for variables in model 2, and fasting blood glucose (mmol/L) and glycated hemoglobin A1c (%).

Adding the cross-product terms in the multivariable model tested the interactions between continuous BMI and sex, age groups, HbA1c, and FBG. To test the robustness of the results obtained from the main analysis, we conducted four sensitivity analyses: excluding participants with high blood pressure (systolic blood pressure ≥ 140 mmHg or diastolic blood pressure ≥ 90 mmHg) [20], with abnormal lipid metabolism (total cholesterol ≥ 5.7 mmol/L or triglyceride ≥ 1.7 mmol/L or low − density lipoprotein cholesterol ≥ 3.4 mmol/L or high − density lipoprotein cholesterol < 0.9 mmol/L for man or high − density lipoprotein cholesterol < 1.0 mmol/L for female) [21], or with decreased eGFR (≤60 mL/min per 1.73 m^2^) [18] or the extreme values(>99^th^ percentile or <1^st^ percentile) at baseline.

## 3. Results

A total number of 6,911 Chinese elderly people (4,110 men and 2,801 women, aged 69 (interquartile range: 67-75) years) were included in the study. The medium of BMI was 24.3 kg/m^2^ (interquartile range: 22.2-26.4 kg/m^2^) (Supplementary Table 1).

Over a mean follow-up of 4.8 ± 0.7 years (full range: 1-5 years), 389 participants (258 men and 131 women) had confirmed DM. The incidence of DM was 5.6% (11.6/1000 person-year). The basic characteristics were presented in Table 1. Comparing those participants with DM in the baseline recruitment, the participants included in the study were with lower level of BMI, FBG, and HbA1c (Supplementary Table 2).

Increased BMI was associated with the risk of DM in the whole groups (Table 2).

Comparing individuals with a BMI range of 18.5-22.5 kg/m^2^ (reference), the hazard ratio for incident DM was 2.13 (95% CI: 1.54~2.95), 2.14 (95% CI: 1.53~3.00), 3.17 (95% CI: 2.19~4.59), and 3.14 (95% CI: 1.94~5.09) for the group with a BMI range of 22.5 to <25.0 kg/m^2^, 25.0 to <27.5 kg/m^2^, 27.5 to <30.0 kg/m^2^, and ≥30.0 kg/m^2^ after baseline age, sex, blood pressure, lipid profiles, and eGFR adjustment (*P* trend < 0.001) (Table 2, model 2). The association between BMI and the risk of DM demonstrated a “straight line” curve (*P* value for nonlinear test = 0.36) (Figure 1). The *P* value for the interaction between continuous BMI and FBG was < 0.001 (Supplementary Table 3). Men (HR = 1.34, 95% CI: 1.20~1.49) tended to have a more robust association between BMI and DM than women (HR = 1.31, 95% CI: 1.13~1.51). Similarly, the younger population (<75 years, HR = 1.32, 95% CI: 1.18~1.48) demonstrated a stronger association compared with the elder participants (≥75 years, HR = 1.31, 95% CI: 1.15~1.49) (Table 3).

The exclusion of participants with high blood pressure (*n* = 3,774), with abnormal lipid profiles (*n* = 3,580), with decreased eGFR (*n* = 394), and the extreme values (>99^th^ percentile or <1^st^ percentile) at baseline (*n* = 839) generated similar results with the prospective analyses (Table 4).

## 4. Discussion

In the current cohort study with 6,911 Chinese aged population, high BMI was found to be associated with high risk of developing DM after adjusting for conventional risk factors of DM such as blood pressure, lipid profiles, and renal function.

Current researches have pointed out that the association between BMI and all-cause mortality follows a “U” [10, 11] or “J” curve [12, 13]. However, the optimal BMI range was based on the relationship between BMI and mortality, not for BMI-DM association. In our study, we found that the association between BMI and the risk of DM demonstrated a “straight line” curve, similar to Hu et al. [22], indicating that the relationship between BMI and DM might follow a different pattern. Bae et al. found that the risk of incident diabetes increased significantly at BMI level of 23 to 24 kg/m^2^ [23]. It is better to be lower in weight even with a normal BMI. The hazard ratio was 2.13 (95% CI: 1.54~2.95) for participants whose BMI was between 22.5 and <25.0 kg/m^2^, compared to those between 18.5 and <22.5 kg/m^2^. The finding is similar to that of Hu et al. [22], who performed a perspective cohort study in a relatively low risk middle-aged and elderly Chinese population. They found that participants with BMI ≥ 22.0 kg/m^2^ (HR = 1.09, 95% CI: 1.09~2.32) had significantly elevated diabetic risk. Chen et al. [24] also found the age-adjusted HR for incident diabetes was 2.51 (95% CI: 2.33~2.70) in overweight individuals (BMI 24.0 to <28.0 kg/m^2^) and 5.58 (95% CI: 5.13~6.07) in obese individuals (BMI of ≥28.0 kg/m^2^), compared with normal-weight individuals (BMI of 18.5 to <24.0 kg/m^2^) in a retrospective cohort study including 211,833 Chinese adults (20–30, 30–40, 40–50, 50–60, 60–70 and ≥70 years old) in 11 cities. These results corroborate the findings of previous epidemiological studies in Europe [25, 26], the U.S.A [27], and Asia [9]. These studies, however, focused on the risks associated with overweight or obesity and were less concerned about the risks associated with a BMI at the lower limit of the normal weight range. Moreover, within the healthy weight range, some proposed an additional breakdown of BMI classification to 18.5-19.9 kg/m^2^, 20.0-22.9 kg/m^2^, and 23.0-24.9 kg/m^2^ [28]. Hence, in order to avoid misperception related to what should be normal or abnormal, the BMI categories in our study differ from those defined by the National Institutes of Health [29] and found that the incident DM with a BMI in upper limit of normal range was higher than that of the lower limit of normal range. To our knowledge, there are no equivalent BMI standards for the elderly. Future studies are needed to determine optimal BMI cut-off for the aged population.

We applied the same BMI classification as used in Ma et al.'s [16] study, but the finding is in contrast to his research. They performed a longitudinal study of 8,735 nondiabetic participants (aged 20-74 years) for a mean follow-up of 6 years. They classified the age into three groups as 20-39, 40-59, and 60-74 years and found that the association between BMI and incident T2DM was extinguished in 60-74-year group (*n* = 1,501). Comparing with participants whose BMI range was between 22.5 and 24.9 kg/m^2^, the hazard risk of incident DM was not significantly different from the group with a BMI range of 18.5-22.4 kg/m^2^ (HR = 0.78, 95% CI: 0.37-1.20), 25.0-27.4 kg/m^2^ (HR = 0.76, 95% CI: 0.47-1.19), 27.5-29.9 kg/m^2^ (HR = 0.87, 95% CI: 0.58-1.28), and ≥30.0 kg/m^2^ (HR = 0.49, 95% CI: 0.21-1.15) after adjusting for sex, age, systolic blood pressure, alcohol use, smoking history, education, regular exercise, family history of diabetes and prediabetes at baseline, and follow-up years. Possible explanations for the inconsistency may lie in difference in BMI reference, diagnosis of DM, sample size, and confounding factors.

Ethnicity might be another reason for the differences in optimal BMI cutoff for the aged population. Accumulating evidences have suggested that the relationship between BMI and body fat deposit differs between ethnic populations [30], and Asians have a higher risk of DM than other ethnic groups even when they were at the same BMI levels [31, 32]. The possible explanation was that Asians have more visceral adiposity than Caucasians, which is more metabolically adverse and contributes to lip toxicity and insulin resistance at any given BMI [30, 33]. Thus, in the Diabetes Prevention Program (DPP) [34], a BMI value of 22.0 kg/m^2^ was selected as the eligibility BMI criteria for Asians and BMI ≥ 23.0 kg/m^2^ was a risk factor for insulin resistance and diabetes in Japanese people [9]. The abovementioned studies might suggest a lower BMI cut-off for Asians compared to Caucasians. The results were consistent with our research. Furthermore, eating habits [35], dietary inflammatory index [36], income status [37], education level [38], and significant heterogeneity in critical metabolic factors [34] might modify the relationship between BMI and DM.

### 4.1. Strengthens and Limitations

The strengthens of the study were a large number of sample size, adjustment of a series of conventional risk factors, and cohort study design. However, some limitations need to be addressed. First, despite considering many possible confounders and performing multivariate analysis, there is a lack of investigation for the correlation of several other risk factors (alcohol consumption, cigarette smoking status, eating habit, and exercise) to BMI and DM. Second, we did not collect information on antidiabetes medications during follow-up, which could result in the loss of new DM case. Third, DM was confirmed by either FGB or HbA1c, but not OGTT, which might lead to misclassification of DM status. We excluded participants with self-reported DM in the follow-up, but undiagnosed DM is still possible [39]. Finally, we did not consider history of diseases in elderly that might affect the incidence of DM.

## 5. Conclusion

Increased BMI was associated with the risk of DM in the Chinese aged population. Thus, it is optimal for the aged population to maintain their body weight within a reasonable range to prevent chronic diseases.

## Figures and Tables

**Table 1:** The basic characteristics of the participants with diabetes mellitus and nondiabetes mellitus in follow-up (*n* = 6,911).

Variables	Diabetes mellitus	Non-diabetes mellitus	Total	*P* value
Samples (*n*)	389	6,522	6,911	—
Female (%)	131, 33.7%	2,670, 40.9%	2,801, 40.5%	0.0048
Age (y)	72 (68, 79)	69 (67, 74)	69 (67, 75)	<0.001
BMI (kg/m^2^)	25.2 (23.2, 27.4)	24.2 (22.2, 26.3)	24.3 (22.2, 26.4)	<0.001
SBP (mmHg)	143 (131, 155)	141 (128, 154)	141 (128, 154)	0.06
DBP (mmHg)	78 (70, 85)	77 (70, 85)	77 (70, 85)	0.35
FBG (mmol/L)	5.82 (5.33, 6.30)	5.25 (4.94, 5.60)	5.29 (4.96, 5.67)	<0.001
HbA1c (%)	6.0 (5.7, 6.2)	5.6 (5.4, 5.8)	5.6 (5.4, 5.8)	<0.001
TC (mmol/L)	4.83 (4.17, 5.55)	5.02 (4.43, 5.70)	5.02 (4.41, 5.69)	<0.001
TG (mmol/L)	1.50 (1.05, 2.14)	1.29 (0.95, 1.81)	1.30 (0.96, 1.82)	<0.001
HDL-C (mmol/L)	1.25 (1.06, 1.54)	1.35 (1.13, 1.62)	1.35 (1.13, 1.62)	<0.001
LDL-C,(mmol/L)	2.78 (2.23, 3.36)	2.99 (2.46, 3.54)	2.98 (2.45, 3.53)	<0.001
eGFR (mL/min per 1.73 m^2^)	83.1 (71.0, 90.7)	84.5 (74.8, 90.9)	84.5 (74.7, 90.9)	0.07

Note: (1) Abbreviation: BMI: body mass index; SBP: systolic blood pressure; DBP: diastolic blood pressure; FBG: fasting blood glucose; HbA1c: glycated hemoglobin A1c; TC: total cholesterol; TG: triglyceride; HDL-C: high-density lipoprotein cholesterol; LDL-C: low-density lipoprotein cholesterol; eGFR: estimated glomerular filtration rate. (2) Abnormal distribution, data were presented as medium plus quartile range.

**Table 2:** The adjusted hazardous ratios and 95% confidence interval of the risk of diabetes mellitus for the baseline BMI groups (*n* = 6,911).

Model	BMI groups							*P* trend
	BMI <18.5	BMI 18.5 to<22.5	BMI 22.5 to<25.0	BMI 25.0 to<27.5	BMI 27.5 to<30	BMI ≥30.0		
Sample	201	1,750	2,130	1,778	777	275	—	—

Case	5	58	130	107	68	26	—	—

Model 1	0.73	1.00 (ref)	2.17	2.17	3.22	3.35	1.34	<0.001
	(0.29~ 1.83)		(1.56~3.00)	(1.56~ 3.02)	(2.25~ 4.62)	(2.09~ 5.31)	(1.23~ 1.45)	

Model 2	0.73	1.00 (ref)	2.13	2.14	3.17	3.14	1.32	<0.001
	(0.29~ 1.82)		(1.54~2.95)	(1.53~3.00)	(2.19~4.59)	(1.94~5.09)	(1.21~ 1.44)	

Model 3	0.77	1.00 (ref)	1.51	1.42	1.83	1.50	1.12	0.01
	(0.31~ 1.92)		(1.09~ 2.10)	(1.01~ 2.00)	(1.26~ 2.66)	(0.92~ 2.44)	(1.03~ 1.22)	

Note: (1) model 1: adjusting age (y) and sex. (2) Model 2: adjusting age (y), sex, systolic blood pressure (mmHg), diastolic blood pressure (mmHg), total cholesterol (mmol/L), triglyceride (mmol/L), low-density lipoprotein cholesterol (mmol/L), high-density lipoprotein cholesterol (mmol/L), eGFR (mL/min per 1.73 m^2^). (3) Model 3: adjusting age (y), sex, systolic blood pressure (mmHg), diastolic blood pressure (mmHg), total cholesterol (mmol/L), triglyceride (mmol/L), low-density lipoprotein cholesterol (mmol/L), high-density lipoprotein cholesterol (mmol/L), eGFR (mL/min per 1.73 m^2^), fasting blood glucose(mmol/L), and glycated hemoglobin A1c (%).

**Table 3:** The adjusted hazardous ratios and 95% confidence interval of the risk of diabetes mellitus in subgroups for the baseline BMI groups (*n* = 6,911).

Group	Model	BMI groups							*P* trend
		BMI <18.5	BMI 18.5 to<22.5	BMI 22.5 to<25.0	BMI 25.0 to<27.5	BMI 27.5 to<30	BMI ≥30.0		
Men	Sample (*n* = 4,110)	117	993	1,282	1,117	450	151	—	—
	Case (*n* = 258)	3	38	84	66	51	16	—	—
	Model	0.60	1.00 (ref)	1.89	1.76	3.45	2.84	1.34	<0.001
		(0.18~ 1.93)		(1.28~ 2.78)	(1.16~ 2.65)	(2.22~ 5.35)	(1.56~ 5.12)	(1.20~1.49)	

Women	Sample (*n* = 2,801)	84	757	848	661	327	124	—	—
	Case (*n* = 131)	2	15	46	41	17	10	—	—
	Model	1.28	1.00 (ref)	2.81	3.31	2.72	4.26	1.31	<0.001
		(0.26~ 4.96)		(1.56~ 5.06)	(1.81~ 6.02)	(1.35~ 5.51)	(1.86~ 9.73)	(1.13~1.51)	

Age (<75 years)	Sample (*n* = 5,177)	116	1,248	1,619	1,388	600	206	—	—
	Case (*n* = 235)	2	28	77	70	40	18	—	—
	Model	0.77	1.00 (ref)	2.08	2.16	3.00	3.67	1.32	<0.001
		(0.18~ 3.23)		(1.35~ 3.23)	(1.38~ 3.39)	(1.80~ 4.84)	(2.00~ 6.76)	(1.18~1.48)	

Age (≥75 years)	Sample (*n* = 1,734)	85	502	511	390	177	69	—	—
	Case (*n* = 154)	3	25	53	37	28	8	—	—
	Model	0.69	1.00 (ref)	2.19	2.08	3.58	2.21	1.31	<0.001
		(0.21~ 2.31)		(1.35~ 3.54)	(1.24~ 3.50)	(2.04~ 6.28)	(0.98~ 5.00)	(1.15~1.49)	

Note: (1) model: adjusting age (y), sex, systolic blood pressure (mmHg), diastolic blood pressure (mmHg), total cholesterol (mmol/L), triglyceride (mmol/L), low-density lipoprotein cholesterol (mmol/L), high-density lipoprotein cholesterol (mmol/L), and eGFR (mL/min per 1.73 m^2^).

**Table 4:** The adjusted hazardous ratios and 95% confidence interval for the risk of diabetes mellitus for the baseline BMI groups: sensitivity analysis.

		BMI groups							*P* trend
		BMI <18.5	BMI 18.5 to <22.5	BMI 22.5 to <25.0	BMI 25.0 to <27.5	BMI 27.5 to <30	BMI ≥30.0		
Sensitivity-1	Sample	129	967	969	732	268	72	—	—
	Case	3	23	48	46	29	5	—	—
	Model	0.81	1.00 (ref)	2.23	2.93	4.81	2.83	1.47	<0.001
		(0.24~2.71)		(1.35~3.69)	(1.75~4.88)	(2.74~8.45)	(1.06~7.56)	(1.28~1.68)	

Sensitivity-2	Sample	137	1,007	977	782	313	115	—	—
	Case	4	33	55	48	25	15	—	—
	Model	0.83	1.00 (ref)	1.86	2.04	2.85	4.00	1.37	<0.001
		(0.29~2.35)		(1.20~2.87)	(1.30~3.21)	(1.68~4.84)	(2.15~7.45)	(1.22~1.54)	

Sensitivity-3	Sample	191	1,658	2,030	1,670	717	251	—	—
	Case	5	47	1118	100	55	23	—	—
	Model	0.82	1.00 (ref)	2.17	2.27	2.93	3.21	1.31	<0.001
		(0.32~2.05)		(1.54~3.05)	(1.59~3.23)	(1.97~4.37)	(1.92~5.35)	(1.19~1.43)	

Sensitivity-4	Sample	95	1,512	1,898	1,596	691	180	—	—
	Case	2	45	113	102	57	11	—	—
	Model	0.72	1.00 (ref)	2.11	2.31	3.02	2.08	1.28	<0.001
		(0.17~2.95)		(1.48~2.98)	(1.61~3.31)	(2.03~4.52)	(1.07~4.05)	(1.17~1.41)	

Note: (1) sensitivity-1: excluding participants whose systolic blood pressure ≥ 140 mmHg or diastolic blood pressure ≥ 90 mmHg (*n* = 3,774). (2) Sensitivity-2: excluding participants whose total cholesterol ≥ 5.7 mmol/L or triglyceride ≥ 1.7 mmol/L or low − density lipoprotein cholesterol ≥ 3.4 mmol/L or high − density lipoprotein cholesterol  < 0.9 mmol/L for man or high − density lipoprotein cholesterol  < 1.0 mmol/L for women (*n* = 3,580). (3) Sensitivity-3: excluding participants whose eGFR ≤ 60mL/min per 1.73 m^2^ (*n* = 394). (4) Sensitivity-4: excluding the extreme values (>99^th^ percentile or <1^st^ percentile) at baseline (*n* = 839). (5) Model: adjusting age (y), sex, systolic blood pressure (mmHg), diastolic blood pressure (mmHg), total cholesterol (mmol/L), triglyceride (mmol/L), low-density lipoprotein cholesterol (mmol/L), high-density lipoprotein cholesterol (mmol/L), and eGFR (mL/min per 1.73 m^2^).

## Data Availability

The reidentified data and SAS code were available upon reasonable request (Renying Xu, email address: xurenying7465@126.com).

## Abbreviations

BMI: Body mass index
DM: Diabetes mellitus
FBG: Fasting blood glucose
HbA1c: Glycated hemoglobinA1c.

## Supplementary Materials

Supplementary Materials Supplementary Table 1: the basic characteristics of the participants (*n* = 6,911). Supplementary Table 2: comparison of baseline characteristics between participants in and out of the analysis (*n* = 9,901). Supplementary Table 3: the interaction analyses between BMI and age, sex, HbA1c, and FBG. Supplementary Figure 1: flow chart of sample recruitment.
