# Supplementary material for: The Relationship between Body Mass Index and Incident Diabetes Mellitus in Chinese Aged Population: A Cohort Study
Source: J Diabetes Res. 2021 Aug 24;2021:5581349. doi: 10.1155/2021/5581349 (PMC8410436; doi:10.1155/2021/5581349)
Supplement: Supplementary Materials — Supplementary Table 1: the basic characteristics of the participants (n = 6,911). Supplementary Table 2: comparison of baseline characteristics between participants in and out of the analysis (n = 9,901). Supplementary Table 3: the interaction analyses between BMI and age, sex, HbA1c, and FBG. Supplementary Figure 1: flow chart of sample recruitment. [file 5581349.f1.doc]

**Supplementary Table 1.** The basic characteristics of the participants (n=6,911)

| Variables | Men | Women | Total | P value |
| --- | --- | --- | --- | --- |
| Samples, n | 4,110 | 2,801 | 6,911 | - |
| Age, y | 70 (67, 76) | 69 (66, 73) | 69 (67, 75) | <0.001 |
| BMI, kg/m^2^ | 24.4 (22.3, 26.4) | 24.1 (22.1, 26.4) | 24.3 (22.2, 26.4) | 0.02 |
| SBP, mmHg | 140 (128, 153) | 142 (129, 155) | 141 (128, 154) | <0.001 |
| DBP, mmHg | 79 (71, 86) | 76 (68, 83) | 77 (70, 85) | <0.001 |
| FBG, mmol/L | 5.30 (4.97, 5.69) | 5.27 (4.95, 5.63) | 5.29 (4.96, 5.67) | 0.16 |
| HbA1c, % | 5.60 (5.40, 5.80) | 5.60 (5.40, 5.90) | 5.6 (5.4, 5.8) | <0.001 |
| TC, mmol/L | 4.84 (4.22, 5.43) | 5.30 (4.70, 5.99) | 5.02 (4.41, 5.69) | <0.001 |
| TG, mmol/L | 1.26 (0.92, 1.77) | 1.36 (1.03, 1.87) | 1.30 (0.96, 1.82) | <0.001 |
| HDL-C mmol/L | 1.27 (1.07, 1.52) | 1.48 (1.25, 1.76) | 1.35 (1.13, 1.62) | <0.001 |
| LDL-C, mmol/L | 2.89 (2.33, 3.40) | 3.15 (2.61, 3.74) | 2.98 (2.45, 3.53) | <0.001 |
| eGFR, mL/min per 1.73 m^2^ | 83.3 (72.3, 89.6) | 86.4 (78.0, 92.5) | 84.5 (74.7, 90.9) | <0.001 |

**Note:**

1. Abbreviation: **BMI,** body mass index; **SBP,** systolic blood pressure; **DBP**, diastolic blood pressure; **FBG**, fasting blood glucose; **HbA1c**, glycated hemoglobin A1c; **TC**, total cholesterol; **TG**, triglyceride; **HDL-C,** high density lipoprotein cholesterol; **LDL-C,** low density lipoprotein cholesterol; **eGFR**, estimated glomerular filtration rate.
2. Abnormal distribution, data were presented as medium plus quartile range

**Supplementary Table 2.** Comparison of baseline characteristics between participants in and out of the analysis (n=9,901)

| Variables | In the study | Out of the study | | Total | P value |
| --- | --- | --- | --- | --- | --- |
|  |  | Diabetes-baseline | Loss to follow up |  |  |
| Samples, n | 6,911 | 2,012 | 979 | 9,901 | - |
| Female, % | 2,801, 40.5% | 732, 36.4% | 420, 42.9% | 3,953, 39.9% | <0.001 |
| Age, y | 69 (67, 75) | 70 (67, 75) | 69 (66,74) | 70 (67,75) | <0.001 |
| BMI, kg/m^2^ | 24.3 (22.2, 26.4) | 25.4 (23.3, 27.7) | 24.4 (22.3, 26.6) | 24.5 (22.4, 26.6) | <0.001 |
| SBP, mmHg | 141 (128, 154) | 146 (133, 160) | 139 (126, 150) | 142 (129, 155) | <0.001 |
| DBP, mmHg | 77 (70, 85) | 77 (70, 85) | 78 (71, 86) | 77 (70, 85) | 0.33 |
| FBG, mmol/L | 5.29 (4.96, 5.67) | 7.30 (6.44, 8.40) | 5.18 (4.88, 5.55) | 5.40 (5.00, 6.03) | <0.001 |
| HbA1c, % | 5.60 (5.40, 5.80) | 6.90 (6.50, 7.70) | 5.60 (5.30, 5.80) | 5.70 (5.40, 6.10) | <0.001 |
| TC, mmol/L | 5.00 (4.41, 5.69) | 4.94 (4.27, 5.62) | 5.04 (4.49, 5.76) | 5.00 (4.39, 5.68) | <0.001 |
| TG, mmol/L | 1.30 (0.96, 1.82) | 1.51 (1.08, 2.16) | 1.27 (0.93, 1.78) | 1.33 (0.98, 1.88) | <0.001 |
| HDL-C mmol/L | 1.35 (1.13, 1.62) | 1.23 (1.04, 1.45) | 1.36 (1.16, 1.62) | 1.32 (1.11, 1.59) | <0.001 |
| LDL-C, mmol/L | 2.98 (2.45, 3.53) | 2.93 (2.36, 3.50) | 3.00 (2.48, 3.56) | 2.97 (2.43, 3.53) | 0.09 |
| eGFR, mL/min per 1.73 m^2^ | 85.3 (74.7, 90.9) | 86.0 (75.4, 92.4) | 88.4 (79.6, 93.4) | 85.4 (75.1, 91.4) | <0.001 |

**Note:**

1. Abbreviation: **BMI,** body mass index; **SBP,** systolic blood pressure; **DBP**, diastolic blood pressure; **FBG**, fasting blood glucose; **HbA1c**, glycated hemoglobin A1c; **TC**, total cholesterol; **TG**, triglyceride; **HDL-C,** high density lipoprotein cholesterol; **LDL-C,** low density lipoprotein cholesterol; **eGFR**, estimated glomerular filtration rate.
2. Abnormal distribution, data were presented as medium plus quartile range.

**Supplementary Table 3****.** The interaction analyses between BMI and age, sex, HbA1c and FBG

| Variables | P Value |
| --- | --- |
| BMI*Age interaction | 0.65 |
| BMI*Sex interaction | 0.72 |
| BMI*HbA1c interaction | 0.51 |
| BMI*FBG interaction | <0.001 |

**Note:**

1. Abbreviation: **BMI,** body mass index; **FBG**, fasting blood glucose; **HbA1c**, glycated hemoglobin A1c.
2. adjusting age (y), sex, systolic blood pressure (mmHg), diastolic blood pressure (mmHg), total cholesterol (mmol/L), triglyceride (mmol/L), low density lipoprotein cholesterol (mmol/L), high density lipoprotein cholesterol (mmol/L), eGFR (mL/min per 1.73 m^2^), fasting blood glucose(mmol/L), glycated hemoglobin A1c (%).


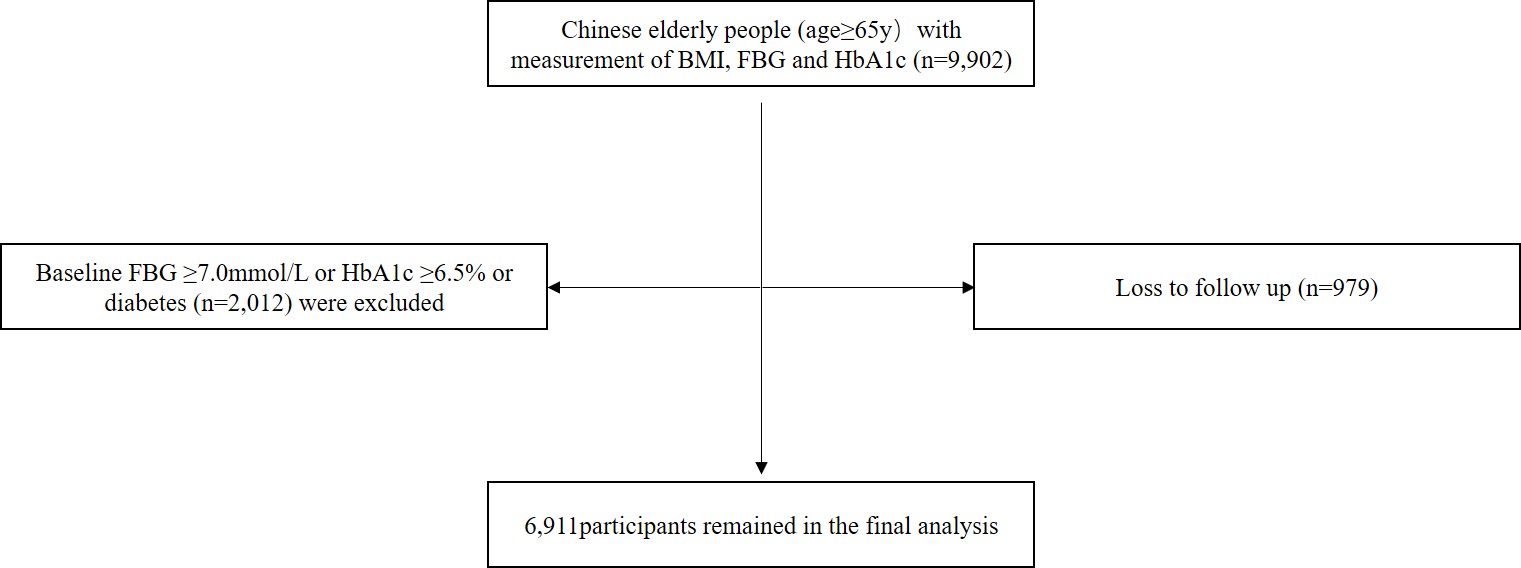


**Supplementary Figure 1****.** Flow chart of sample recruitment

Abbreviation: **BMI,** body mass index; **FBG**, fasting blood glucose; **HbA1c**, glycated hemoglobin A1c.
